# Crack Initiation in Compacted Graphite Iron with Random Microstructure: Effect of Volume Fraction and Distribution of Particles

**DOI:** 10.3390/ma17133346

**Published:** 2024-07-06

**Authors:** Xingling Luo, Konstantinos P. Baxevanakis, Vadim V. Silberschmidt

**Affiliations:** Wolfson School of Mechanical, Electrical and Manufacturing Engineering, Loughborough University, Loughborough LE11 3TU, UK; x.luo4@lboro.ac.uk (X.L.); k.baxevanakis@lboro.ac.uk (K.P.B.)

**Keywords:** compacted graphite iron, damage mechanisms, random microstructure, numerical model, distribution of inclusions

## Abstract

Thanks to the distinctive morphology of graphite particles in its microstructure, compacted graphite iron (CGI) exhibits excellent thermal conductivity together with high strength and durability. CGI is extensively used in many applications, e.g., engine cylinder heads and brakes. The structural integrity of such metal-matrix materials is controlled by the generation and growth of microcracks. Although the effects of the volume fraction and morphology of graphite inclusions on the tensile response of CGI were investigated in recent years, their influence on crack initiation is still unknown. Experimental studies of crack initiation require a considerable amount of time and resources due to the highly complicated geometries of graphite inclusions scattered throughout the metallic matrix. Therefore, developing a 2D computational framework for CGI with a random microstructure capable of predicting the crack initiation and path is desirable. In this work, an integrated numerical model is developed for the analysis of the effects of volume fraction and nodularity on the mechanical properties of CGI as well as its damage and failure behaviours. Finite-element models of random microstructure are generated using an in-house Python script. The determination of spacings between a graphite inclusion and its four adjacent particles is performed with a plugin, written in Java and implemented in ImageJ. To analyse the orientation effect of inclusions, a statistical analysis is implemented for representative elements in this research. Further, Johnson–Cook damage criteria are used to predict crack initiation in the developed models. The numerical simulations are validated with conventional tensile-test data. The created models can support the understanding of the fracture behaviour of CGI under mechanical load, and the proposed approach can be utilised to design metal-matrix composites with optimised mechanical properties and performance.

## 1. Introduction

Cast irons are usually categorized into different groups based on their microstructure due to the crucial effect of their microstructural features [1]. Compacted graphite iron (CGI) is broadly used in the automotive industry, for example, in brakes, exhaust manifolds, cylinder heads, and cylinder blocks, thanks to its good combination of thermal conductivity and mechanical properties [2,3]. The volume fraction, shape, size, and spacing of graphite particles in CGI significantly affect its mechanical properties and fracture mechanism [4,5]. Changes in chemical composition are insufficient to meet specific requirements to improve engine performance. The most efficient approach is to modify the microstructure of cast-iron materials, thereby enhancing their performance and extending their operational lifespan.

As graphite particles are the weakest of CGI’s main constituents, cracks always tend to initiate inside such inclusions or at the interface between them and the matrix [6,7]. With scanning electron microscopy (SEM), Hieber [8] found that fractures usually occurred within graphite flakes as well as at their interfaces. It was concluded that the fracture patterns in both particles and the matrix were unaffected by the strain rate, as similar features were observed for both traditional tensile tests and impact loading. Lopez-Covaleda et al. [9] investigated crack initiation by using a semi-in situ technique. They found that a cyclic plastic zone of graphite particles at interfaces exceeded that for bulk particles. This could explain the enhancement of crack initiation by the presence of graphite clusters. Yang et al. [10] established that the stress–strain distribution in a cutting region of CGI was affected by graphite particles with different sizes and distributions. Tensile strength, fracture toughness, and impact properties of CGI were evaluated by Gregorutti et al. [11] for various microstructures. Still, experimental analysis of CGI cannot predict the strain at the damage onset for each graphite particle, thus lacking a deep insight into its fracture behaviours. Furthermore, the effects of particle spacing on effective plastic strain and equivalent stress during the deformation are hard to obtain experimentally. At the same time, since the crack growth rate of CGI is high, it is difficult to capture details of the fracture process with SEM. Hence, numerical methods can be used to complement experimental studies. Generally, there are two main approaches used in continuous microstructure-based numerical models: representative volume elements (RVEs) [12,13,14] and unit cells [15,16]. There are three main types of micromechanical methods used for cast irons: (i) direct introduction of real microstructure obtained from SEM or CT (computed tomography) images [17]; (ii) utilisation of idealized shapes of graphite particles with their regular arrangement [18]; and (iii) modelling based on random distribution of particles (e.g., to capture the mechanical response or fracture response by considering the interactions between inclusions [19]). 

Modelling based on the microstructure is a crucial initial step for the simulation of the mechanical properties of materials. The next step is the introduction of damage into numerical schemes. Cohesive zone models (CZMs) have been extensively employed to analyse debonding between the matrix and particles as well as crack growth in CGI at the micro level, especially with the unit cell approach [20]. A Johnson–Cook damage model could also predict the initiation and propagation of cracks in CGI with less computational effort and good predictions of the crack path for realistic graphite morphology [21]. Yang et al. [22] developed a simple but effective approach for a relation between graphite morphology and the mechanical properties of CGI. They also found that 2D slices of a 3D graphite microstructure can reasonably depict the spatial morphology of CGI. The effect of graphite particle size, orientations, and volume fraction on tensile properties was investigated by Zhang et al. [23], which revealed a quantitative relation between the tensile properties of CGI and graphite morphology. 

However, the influence of the distance between graphite particles on cracks was not considered, although the interaction between inclusions can cause stress concentration in the matrix. This occurs mostly in areas bridging the neighbouring particles, accelerating the crack-propagation process in them. The narrow matrix bridges facilitate the concentration of high stress [17]. Palkanoglou et al. [18] investigated the interaction between two neighbouring particles with a 2D unit cell with two graphite inclusions. He et al. [24] considered a microstructure with circular particles of different sizes randomly distributed in the matrix. Yang et al. [10] generated four microstructural models with various sizes and distributions of particles. Their findings revealed that such microstructures affected stress–strain distributions in the material. However, only a circular shape of graphite particles was used in their models.

In this paper, the interaction between a graphite particle and its nearest neighbour as well as with the four closest particles is analysed in terms of respective distances. The effect of such distances on crack-initiation strain is studied numerically, also considering the effects of the size and orientation of inclusions. The focus is mainly on the effect of nodularity, the distance between graphite particles, and their orientation in CGI. Analysis was limited to graphite particles with an aspect ratio below 10:1, which is suitable for most inclusions in CGI (the effect of higher aspect ratios will be studied separately). The random microstructure-based models were generated using an in-house Python script to achieve particle distributions close to the real ones. The elastoplastic behaviour and cracking of the matrix, along with decohesion at the graphite–matrix interface and particle fracture, are simulated using the Johnson–Cook damage model. These findings contribute to understanding the fracture mechanism of CGI.

## 2. Methodology

### 2.1. Model Geometry and Boundary Conditions

In this section, a new numerical approach is proposed to investigate the effects of volume fraction and nodularity on the Young’s modulus, damage, and failure behaviours of CGI. Compacted graphite iron EN-GJV-450 was used in this study; its chemical composition is given in Table 1. 

One of the main characteristics of the morphology of graphite particles in CGI is nodularity, which is calculated for a sample with the following equation:

(1) $$Nodularity=\frac{\sum S_{a}}{\sum S_{a}+\sum S_{b}}\times 100\%,$$

where $S_{a}$ and $S_{b}$ are the areas of round and elongated particles, respectively. The random microstructure-based finite-element models were generated with the Python script. The model has a square shape with a side length of 500 µm, closely aligning with the geometric parameters obtained in experiments (see Table 2) [18]. The numerical simulations disregarded very small particles to avoid excessive computational efforts. This study used smoothened (elliptical) shapes for particles; direct introduction of complex shapes of vermicular and graphite flake inclusions from real-life microstructures would significantly increase the statistical variability of calculated results. So, the effect of the real geometry of inclusions is studied elsewhere. 

The determination of the distance between a particle and its adjacent four particles (nearest neighbours) was carried out with the ND plugin, written in Java (SE 7) and implemented in Image J (1.53K) [26] (all particles had smooth elliptical shapes). Its basic algorithm is as follows:

(i) The centroid coordinates of each particle (x, y) are derived from the results table of ImageJ.
(ii) The distance between a pair of particles i and j is calculated as
  (2) $$d=\sqrt{{\left(y_{j}-y_{i}\right)}^{2}+{\left(x_{j}-x_{i}\right)}^{2}},$$

**Figure 1:**
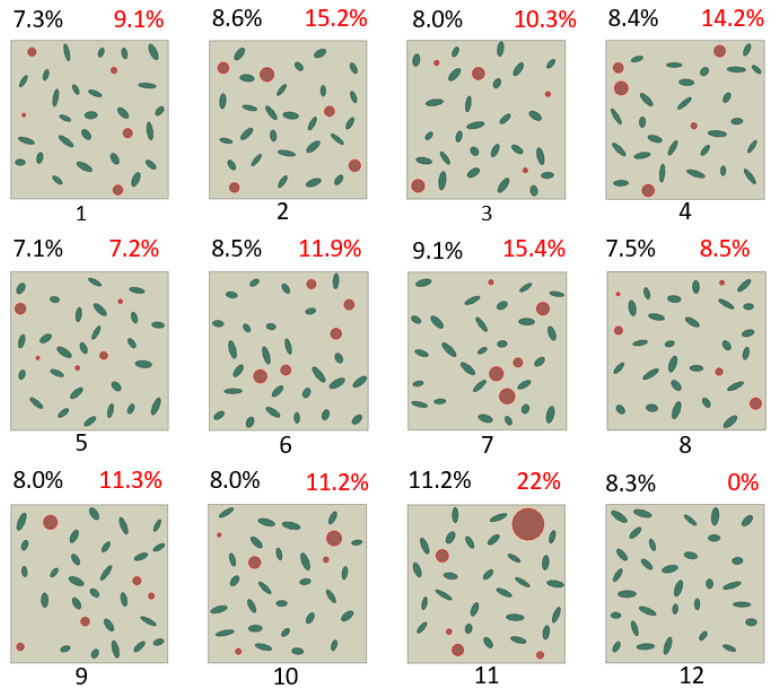
Generated random microstructures of CGI with graphite inclusions for analysis of particle interaction. (The graphite volume fraction is in black numbers, while nodularity is in red numbers. Circular particles are denoted in red.) Numbers 1–12 denote 12 different FEM models.

Twelve cases of random graphite particles were generated for analysis (see Figure 1). The fractions of each microconstituent’s domain were calculated by counting the number of pixels corresponding to the respective colours. The volume fraction of graphite in these models ranged from 7.1% to 11.2%. More model details are presented in Table 3.

**Figure 2:**
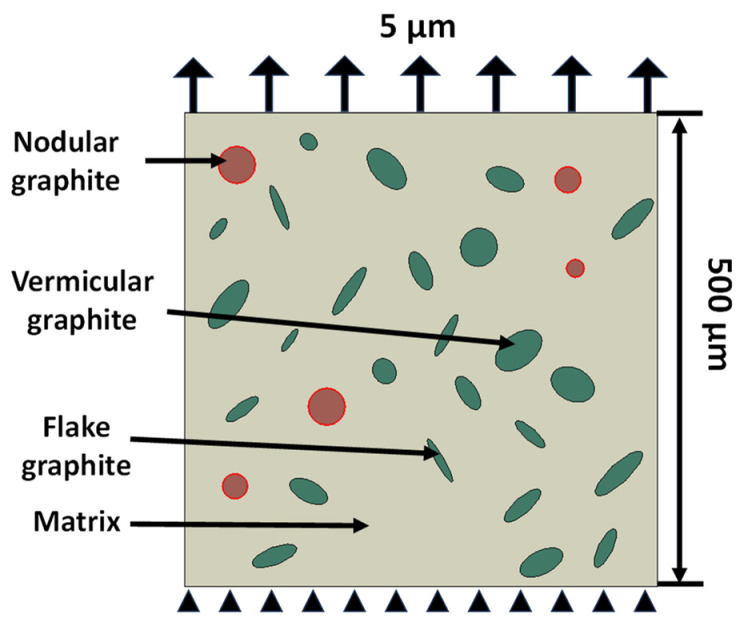
FE model of CGI with loading and boundary conditions. (*Nodular* refers to circular graphite particles while *vermicular* and *flake* inclusions are represented by thick and thin ellipses, respectively).

In simulations, the lower edge of the 2D FE models was constrained in both the x and y directions while the upper edge was exposed to a uniform displacement $u_{y}$ of 5 µm (see Figure 2). In the numerical simulations, the model was meshed with linear constant-strain triangular elements (CPE3). 

### 2.2. Statistical Validation of Graphite Particle Orientation

**Figure 3:**
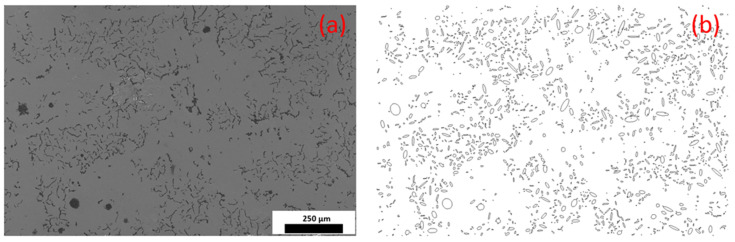
Microstructure morphology of CGI: (**a**) SEM image; (**b**) image processed with ImageJ.

**Figure 4:**
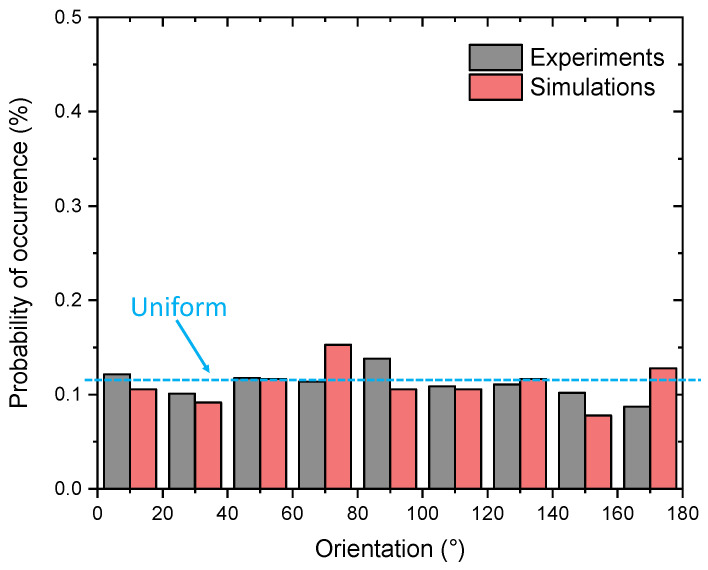
Probability of occurrence of graphite particles at different angles.

The digital image of the microstructure morphology of CGI was generated from SEM as shown in Figure 3a. The complex shapes of graphite particles were processed with ImageJ software to obtain simplified elliptical shapes of graphite and determine their orientation (Figure 3b). Particles with a diameter of less than 20 µm were excluded from our statistical analysis. In this study, the rotation angles of 1019 graphite particles were analysed from SEM images, along with 360 graphite particles from 12 developed models (Figure 1). For 9 bands of orientation angles, the probability density for the orientation of graphite particles was around 11.1% at the microstructure level, indicating a close to uniform distribution (Figure 4).

### 2.3. Constitutive Relations

Johnson–Cook’s constitutive model was first proposed in 1983 to analyse large plastic deformations and fracture behaviour of materials [27]. It describes the plastic behaviour of the material and accounts for the effects of the strain rate, temperature, and large plastic strains on the yield point. The metallic matrix and graphite particles were considered elastoplastic and isotropic. The matrix constituents were described using the Johnson–Cook constitutive model expressed as [28]

(3) $$\sigma =(A+B\varepsilon^{n})\left[1+C\ln\frac{{\dot{\bar{\varepsilon }}}^{pl}}{{\dot{\bar{\varepsilon }}}_{0}^{pl}}\right]\left(1-{\hat{\theta }}^{m}\right),$$

where A, B, and n are the yield stress at zero strain, hardening coefficient, and strain hardening index, respectively. However, for the studied case with isothermal conditions and low strain-rate sensitivity, the last two respective terms in this model are reduced to unity. Hence, the yield stress based on Equation (3) can be expressed as

(4) $$\bar{\sigma }=A+B{\left({\bar{\varepsilon }}^{pl}\right)}^{n}.$$

Failure is considered to occur when the damage parameter, *ω*, defined as

(5) $$\omega =\sum \left(\frac{∆{\bar{\varepsilon }}^{pl}}{{\bar{\varepsilon }}_{f}^{pl}}\right),$$

exceeds 1 (here, $∆{\bar{\varepsilon }}^{pl}$ is an increment of the equivalent plastic strain). The strain at failure is presented as

(6) $${\bar{\varepsilon }}_{f}^{pl}=\left[d_{1}+d_{2}exp\left(d_{3}\frac{p}{q}\right)\right]\cdot \left[1+d_{4}\ln\dot{\varepsilon }\right]\cdot \left[1+d_{5}\frac{T-T_{R}}{T_{m}-T_{R}}\right],$$

where $d_{1}$–$d_{5}$ are the failure parameters measured at or below the transition temperature. p is the hydrostatic stress (the average of the three normal principal stresses), q is the equivalent von Mises stress, $T_{R}$ is the room temperature, and $T_{m}$ is 1600 °C. As discussed, for the analysed case, $d_{4}=0$ and $d_{5}=0.$ The set of Johnson–Cook’s parameters used in the model was adopted from experimental tests conducted by Razanica et al. (see Table 4) [29]. 

The parameters describing the mechanical behaviour of graphite inclusions that were used in simulations are provided in Table 5.

### 2.4. Convergence Study and Model Validation

**Figure 5:**
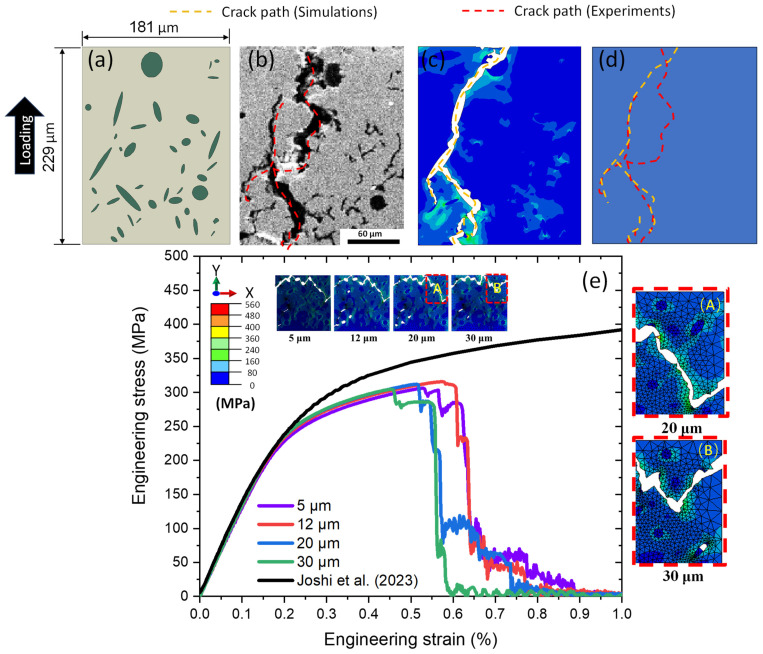
Microstructural morphology and corresponding numerical results for tensile loading: (**a**) sketch of numerical model; (**b**) microstructure after tensile test; (**c**) numerical results; (**d**) comparison of experimental crack path (red) and simulated one (orange); (**e**) experimental and calculated tensile engineering stress–strain curves together with crack path for CGI obtained with different FE mesh sizes; (**A**) zoomed-in view for 20 µm mesh size; (**B**) zoomed-in view for 30 µm mesh size (experimental stress–strain data are from [31]).

For validation, a CGI specimen was subjected to tensile loading with a displacement rate of 0.6 mm/min. The reconstructed geometry (Figure 5a) was generated based on a real microstructure of CGI employing the same approach based on smoothened particle shapes as described above; the microstructures of CGI after the test are shown in Figure 5b. Since a simplified graphite morphology was employed, the model cannot reproduce the real-life crack path in all its details. Still, the predicted crack path (Figure 5c) showed a good agreement with the experimental data (Figure 5d).

The FE mesh of 20 µm was applied to the microstructure for accurate predictions of the damage behaviour and failure models. It was obtained by analysis of one of the models with the generated random microstructure. Four different mesh sizes—5, 12, 20, and 30 µm—were employed to assess the convergence of the numerical results. The predicted engineering stress–strain curves for CGI with a volume fraction of 8.0% are plotted in Figure 5e together with the crack paths calculated for various mesh sizes. It is noted that the crack path did not change much for the first three mesh sizes. However, the crack path changed when the mesh size was set to 30 µm (compare Figure 5A,B). Hence, the 20 µm mesh size was selected for all forthcoming simulations for the accuracy of results and lower computational cost. 

## 3. Results and Discussion

### 3.1. Effect of Volume Fraction and Nodularity

**Figure 6:**
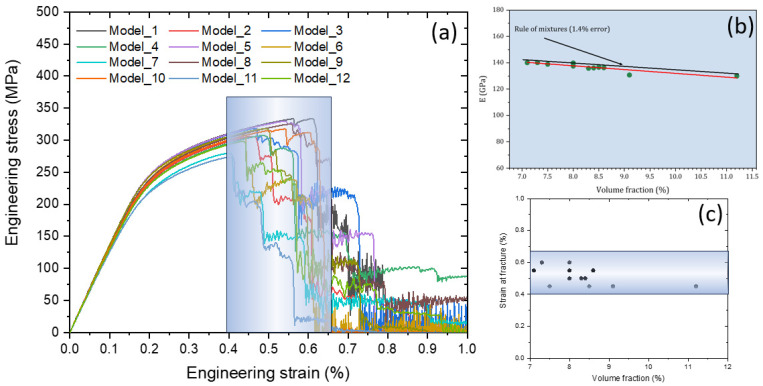
Effect of volume fraction of graphite inclusions: (**a**) calculated engineering stress–strain curve of CGI; (**b**) comparison between calculated Young’s modulus and theoretical values based on rule of mixture (red—simulations, black—rule of mixtures); (**c**) relationship between strain at fracture and volume fraction.

The developed 2D models were employed to explore the effect of volume fraction and nodularity on the mechanical properties of CGI, the onset of each failure mode, and the crack paths. As discussed, the graphite particles were randomly distributed in the matrix, following distributions of their size and perimeter as well as the volume fraction, using a reasonable range for these parameters based on statistical experimental data (see Table 2). The simulation results for engineering stress–strain curves for 12 models are shown in Figure 6a. As a result of the introduction of soft graphite particles into the hard matrix, an increase in the volume fraction of inclusions resulted in a decrease in the Young’s modulus that followed the rule of mixture (note a good agreement between the simulated data and the theoretical values in Figure 6b). The graphite volume fraction in CGI was in the range from 7.1% to 11.2%, leading to a decrease in the effective Young’s modulus of the cast iron from some 140 GPa to 130 GPa. This increase in the volume fraction also diminished the predicted yield strength and the ultimate tensile strength, but the level of strain at failure remained nearly the same: all crack-propagation stages happened within the strain range of 0.4% to 0.64% (the blue region in Figure 6c that corresponds to sharp drops in the stress levels in Figure 6a). Such drops reflected the crack formation accompanied by the creation of local unloaded zones due to the shielding effect. 

**Figure 7:**
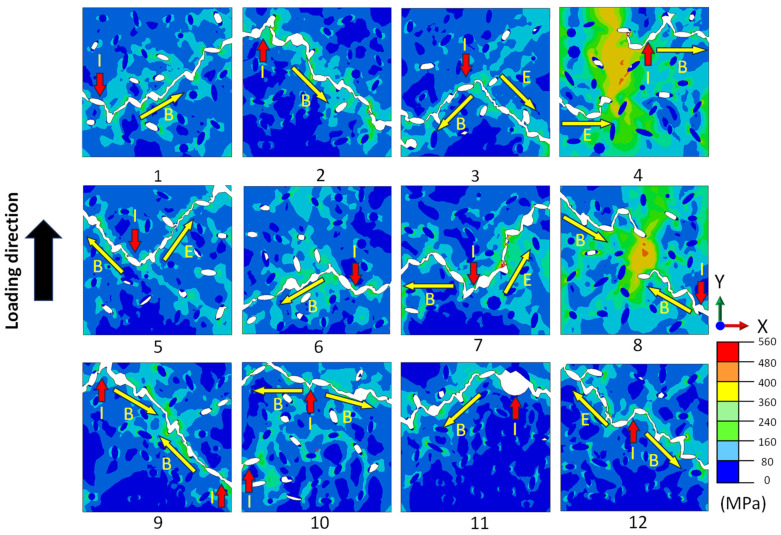
Crack propagation and distribution of von Mises stress for 12 models with random microstructures of CGI (see Figure 1): red arrow I—crack initiation point; yellow arrow B—beginning of crack propagation; yellow arrow E—end of crack propagation.

**Figure 8:**
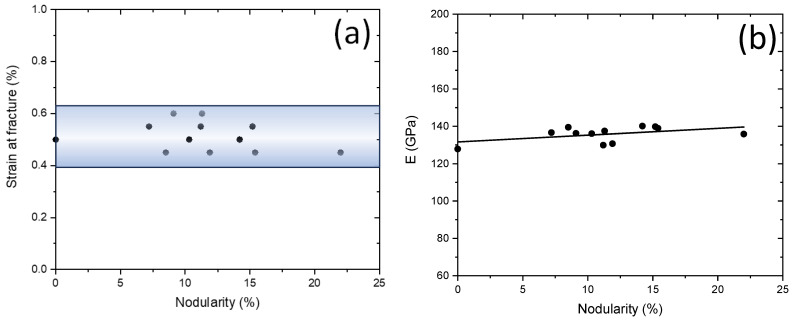
Effect of nodularity on strain at fracture (**a**) and Young’s modulus (**b**) for 12 models with random distributions of graphite.

The crack path and the distribution of von Mises stress in CGI for different distributions of graphite particles are depicted in Figure 7. The crack was initiated when the damage parameter ω under tension exceeded one. The stress concentration and cracks tend to appear in two closely located graphite particles (separated by narrow matrix ligaments) or near larger-sized inclusions (indicated by red arrows in Figure 7). Apparently, the cracks predominantly initiated between two graphite flake particles due to their high local stress concentration. In the case of a sufficiently large distance between two neighbouring graphite particles, crack formation was unlikely even in the presence of high-stress concentration (see Figure 7, model 8). The generated main crack propagated mostly in the matrix, from one inclusion to the neighbouring one, until the specimen was fully fragmented by the crack. Further investigation on the effect of nodularity on the tensile property and fracture behaviour of CGI was conducted employing the developed models with random microstructures. The relationship between the strain at fracture and nodularity is presented in Figure 8a. It was found that the nodularity only insignificantly affected the crack initiation strain, consistent with Figure 6c. In contrast, analysis of the obtained numerical results demonstrated that the Young’s modulus increased with growing nodularity (Figure 8b), which is consistent with the simulations conducted by Yang et al. [22]. This is mainly due to a more even stretching of the matrix when graphite morphology is more uniform. These observations agree with the impact of graphite content and morphology on the properties of CGI.

### 3.2. Effect of Graphite Particle Spacing

**Figure 9:**
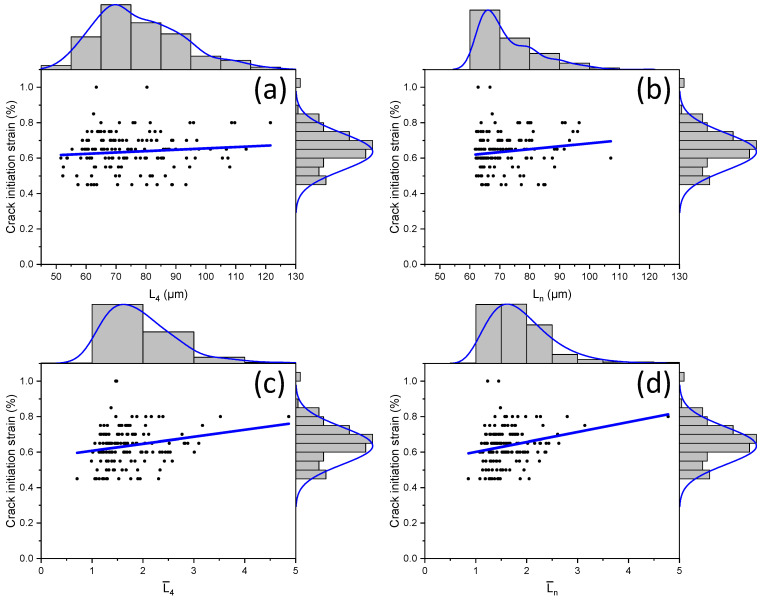
Crack initiation strain in damaged graphite particles for 12 models with random microstructures: (**a**,**b**) without considering size effect; (**c**,**d**) considering size effect. (The blue lines are the trend lines for the clouds of black dots).

In previous SEM studies and simulations [9], the distance between graphite particles and their size significantly influenced the strain at fracture. However, the size effect in fracture evolution was not considered. Therefore, the influence of distance to the nearest neighbours of a graphite particle and its size on damage evolution is investigated in this section. The average distances of each particle to its four neighbours ($L_{4}$) and to the nearest neighbour ($L_{n}$) were evaluated and used for analysis together with their normalized quantities with respect to the major axis’s length particle (see the employed notation in Table 6). The fracture behaviour obtained in simulations and respective statistical results are summarised in Figure 9. Each black dot represents the strain at which the crack occurred in a graphite particle, together with the distance between the cracked graphite particle and its nearest neighbour. Apparently, cracks predominantly occurred when this distance was in the range of 60–70 μm and the macroscopic (global) strain was between 0.6% and 0.7% (Figure 9b). Clearly, the range in distance for the four nearest neighbours was higher—50–122 μm.

Additionally, as the distance between the graphite particles increased, the strain at which the crack appeared also grew. This trend became more pronounced when considering the size of the graphite particle (Figure 9c). Here, the axis of abscissas was determined by the ratio of the distance between a graphite particle and its nearest neighbour to the size of the particle itself. Crack initiation was found to predominantly occur for the normalised distance in the range between one and two. The data for normalised distances are more clustered (Figure 9c,d). So, accounting for the size of graphite particles not only enhances the positive correlation between the crack initiation strain and distance between inclusions but also narrows the range of such distances. It was also noted that all four trend lines indicate that the lower the distance between graphite particles, the earlier the crack initiation. 

### 3.3. Effect of Graphite Particle Orientation

**Figure 10:**
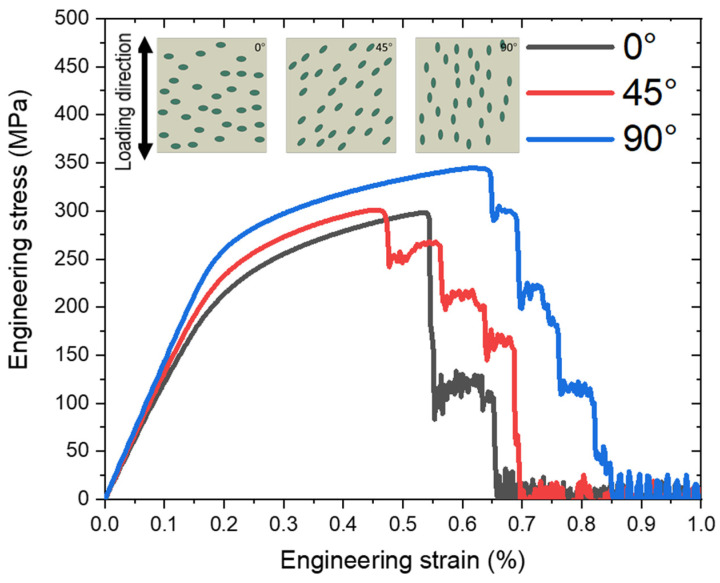
Tensile behaviour of CGI with random distributions of graphite particles for their three different orientations (the angle is measured from the horizontal line, perpendicular to the loading direction).

The influence of the orientation of graphite particles on the mechanical behaviour of, and fracture evolution in, CGI was investigated. The aim of this study was to use the theoretical model to explore the potential impact of graphite particle orientation in a controlled manner to better understand its effects. The orientation effect on engineering stress–strain curves for samples of the same size and with the same volume fraction of graphite of 8.3%, presented in the form of the particles with the same dimensions and shape (aspect ratio of 2), is depicted in Figure 10. It demonstrates that as the angle increased, the effective Young’s modulus of CGI and its overall strength increased (for the latter, the increase between cases of 0° and 45° was rather small). The macroscopic stiffness value reflects the respective changes (i.e., rise) in the effective cross-section of the matrix domain (not occupied by graphite) with a much higher Young’s modulus of this constituent. As established in simulations, it was easier for the crack to initiate when the particles were positioned at 45° to the loading direction. In such orientation, during the loading, they were exposed to high values of both tensile and shear stresses. The details of crack initiation strain for particles with different orientations are briefly discussed in the Appendix A. 

**Figure 11:**
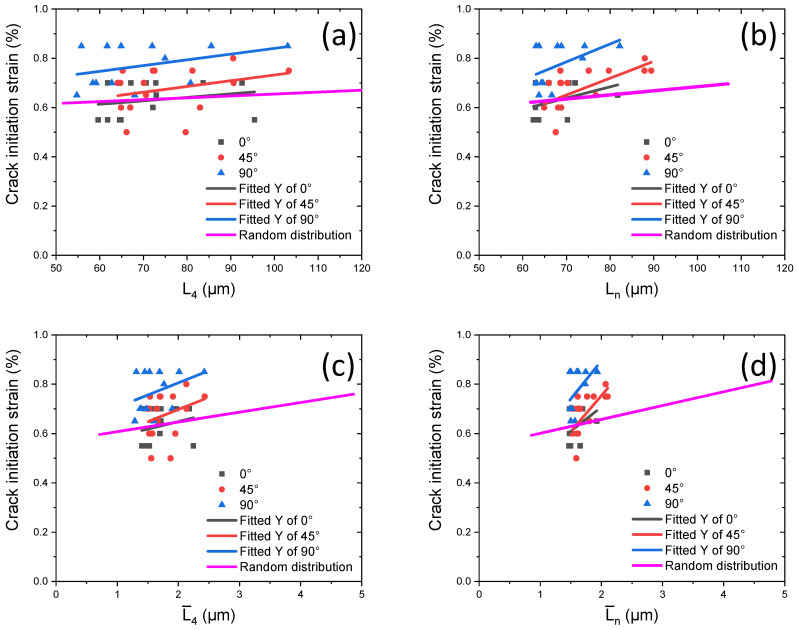
Crack initiation strain for three studied cases of particle orientations in comparison with microstructure with random orientations of inclusions: (**a**,**c**) without considering size effect; (**b**,**d**) considering size effect. (The pink line represents the results obtained for the random orientations; see Figure 9).

These data are compared for three studied cases of orientations as well as with the data obtained for microstructures with random distributions of particles and their orientations in Figure 11. The inclination angle of particles directly affected the crack initiation strain: for 90°, it was around 0.7–0.85% (blue lines in Figure 11), for 45°, it was 0.62–0.75% (red lines), and for 0°, it was 0.55–0.62% (black lines). This indicates that the model with vertical graphite particles exhibited the best crack resistance for this type of loading. The case with the inclination angle of particles at 45° demonstrated not only the early onset of particle cracking but also its considerable duration in terms of strain (as compared to 0°), as reflected in the stress–strain curve in Figure 10. 

The introduction of particles with varying orientations resulted in general deterioration of their performance, close to that of the worst case with the fixed orientation (i.e., horizontal inclusions). In addition, the influence of the distance to the nearest inclusion on the crack was stronger than that of the average distance to four neighbours (compare Figure 11a,b). Meanwhile, considering the size effect (i.e., transition to normalized distances) makes these trends more pronounced (compare Figure 11a with Figure 11c or Figure 11b with Figure 11d), indicating rapid crack occurrence in larger graphite particles that correspond to the phenomena observed in our experiments.

## 4. Conclusions

In this paper, FE models with a random distribution of graphite particles in the metallic matrix of cast iron were developed and implemented to investigate and quantify the effects of different microstructural parameters on the damage and fracture behaviours of CGI. Interface debonding was not included into the model due to the lack of experimental data on the underpinning mechanical processes and a high computational cost for such analysis. The main conclusions are as follows:

1. The present approach effectively captures the mechanical and fracture behaviours of CGI, as evidenced by the comparison of the crack path and effective stress–strain curves between the experimental data and numerical results.
2. The macroscopic Young’s modulus of CGI increased with the increase in the nodularity and the volume fraction of graphite particles.
3. Crack initiation strain demonstrated a complex dependency on the aspect ratio, orientation, and distribution of graphite inclusions, with the particle size playing a significant role. Accounting for the size of the graphite particles helps us to understand the damage and fracture mechanisms of CGI.

These observations could be useful for the production of modern cast irons as well as for the better understanding of damage and fracture in metal-matrix composites.

## Figures and Tables

**Table 1:** Chemical composition (%) of EN-GJV-450 [25].

**Fe**	**C**	**Si**	**Cu**	**Mn**	**Sn**
92	3.8	2.42	0.917	0.297	0.083
**Cr**	**Ni**	**S**	**P**	**Ce**	**Mg**
0.025	0.023	0.013	0.01	0.01	0.009
**Al**	**Ti**	**V**	**Mo**	**Nb**	
0.008	0.009	0.008	0.001	0.002	

**Table 2:** Statistical values of geometrical parameters for graphite particles in CGI.

	Graphite Fraction (%)	Perimeter (μm)	Area (μm^2^)
Experiments [18]	5.2–11.37	3.54–315.88	0.99–6086.96
Simulations	7.1–11.2%	31.4–263.76	78.5–5538.96

**Table 3:** Model details.

Software	Analysis Type	Domain Length	Graphite (%)	Mesh Size	Element Type	Loading
Abaqus (2021)	Dynamic explicit	0.5 mm	7.1–11.2%	20 µm	Triangular CPE3	Tensile

**Table 4:** Calibrated material and failure-model parameters from [29].

*E* (GPa)	*ν* (–)	*A* (MPa)	*B* (MPa)	*n*	*d*_1_	*d*_2_	*d*_3_
152.5	0.26	260	1010	0.467	0.0133	0.0645	8.057

**Table 5:** Constitutive parameters for graphite [18,30].

Mass Density (tonne/mm³)	Young’s Modulus (GPa)	Poisson’s Ratio
2.26 × 10^−9^	15.85	0.2
Plastic strain	Stress (MPa)	
0	27.56	
0.000512	27.62	
0.00102	27.76	
0.00205	28.32	
0.00307	29.25	
0.00409	30.53	
0.00512	32.17	
0.00614	34.18	

**Table 6:** Notation for analysis of effect of distance between graphite particles.

$L_{4}$	Average distance from four neighbours (μm)
$L_{n}$	Nearest neighbour distance (μm)
$\bar{L_{4}}$	Average distance from four neighbours/normalised with major axis of particle
$\bar{L_{n}}$	Nearest neighbour distance/normalised with major axis of particle

**Figure A1:**
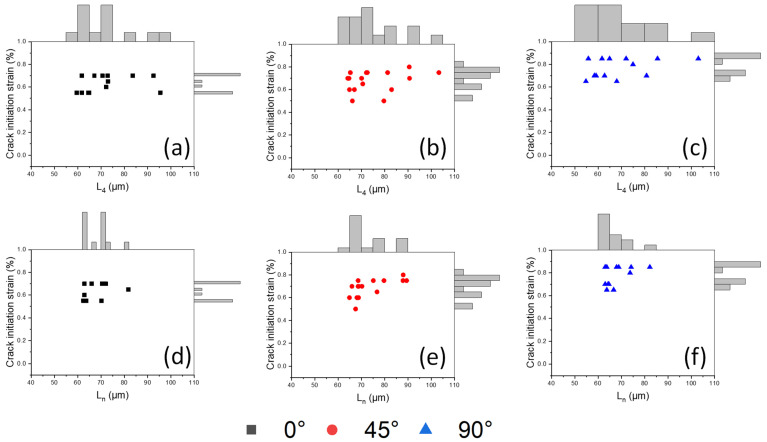
Comparisons of crack initiation strain and distances between inclusions for different orientations of graphite particles: (**a**,**d**) 0°; (**b**,**e**) 45°; (**c**,**f**) 90° (the angle is measured from the horizontal line, perpendicular to the loading direction).

## Data Availability

The original contributions presented in the study are included in the article, further inquiries can be directed to the corresponding author.

## Appendix A. Effect of Graphite Particle Orientation

To demonstrate the effect of graphite particle orientation on the damage and fracture of CGI, three models with 30 graphite particles with a fixed volume fraction of 8.3% and the same size of graphite particles were investigated (see Figure 10). Each datapoint in Figure A1 correspond to a crack initiation strain for a single graphite particle with respective distances to the nearest neighbour and four nearest neighbours.

## References

[1] König M. (2010). Literature review of microstructure formation in compacted graphite Iron. Int. J. Cast Met. Res..

[2] Cao M., Baxevanakis K.P., Silberschmidt V.V. (2023). High-temperature behaviour and interfacial damage of CGI: 3D numerical modelling. Multiscale Multidiscip. Model. Exp. Des..

[3] Li J., Wang P., Cui X., Li K., Yi R. (2013). Gray cast iron cylinder head thermal mechanical fatigue analysis. Proceedings of the FISITA 2012 World Automotive Congress.

[4] Zhang Y., Pang J., Shen R., Qiu Y., Li S., Zhang Z. (2018). Investigation on tensile deformation behavior of compacted graphite iron based on cohesive damage model. Mater. Sci. Eng. A.

[5] Liu Y., Li Y., Xing J., Wang S., Zheng B., Tao D., Li W. (2018). Effect of graphite morphology on the tensile strength and thermal conductivity of cast iron. Mater. Charact..

[6] Mohammed W.M., Ng E., Elbestawi M.A. (2011). Modeling the effect of the microstructure of compacted graphite iron on chip formation. Int. J. Mach. Tools Manuf..

[7] Luo X., Baxevanakis K.P., Silberschmidt V. (2024). V Microstructure-based CZE model for crack initiation and growth in CGI: Effects of graphite-particle morphology and spacing. Solids.

[8] Hieber A.F. (1978). Fracture in compacted graphite iron. AFS Trans..

[9] Lopez-Covaleda E.A., Ghodrat S., Kestens L.A.I. (2020). Semi in-situ observation of crack initiation in compacted graphite iron during thermo mechanical fatigue. Int. J. Fatigue.

[10] Yang H.W., Wang X.M., Liu W., Huang W., Wu M., Xue M.L., Li L. (2024). Influence of distribution and size of graphite particle on the machinability of nodular cast iron. Eng. Fract. Mech..

[11] Gregorutti R.W., Grau J.E. (2014). Mechanical properties of compacted graphite cast iron with different microstructures. Int. J. Cast Met. Res..

[12] Hollister S.J., Kikuchi N. (1994). Homogenization theory and digital imaging: A basis for studying the mechanics and design principles of bone tissue. Biotechnol. Bioeng..

[13] Okereke M., Keates S., Okereke M., Keates S. (2018). Boundary Conditions. Finite Element Applications: A Practical Guide to the FEM Process.

[14] Luo X., Baxevanakis K.P., Silberschmidt V.V., Li S. (2024). Tensile deformation of compacted graphite iron with realistic microstructures: Effect of morphology of graphite inclusions. Computational and Experimental Simulations in Engineering.

[15] Andriollo T., Thorborg J., Tiedje N.S., Hattel J. (2015). Modeling of damage in ductile cast iron—The effect of including plasticity in the graphite nodules. IOP Conf. Ser. Mater. Sci. Eng..

[16] Andriollo T., Thorborg J., Tiedje N., Hattel J. (2016). A micro-mechanical analysis of thermo-elastic properties and local residual stresses in ductile iron based on a new anisotropic model for the graphite nodules. Model. Simul. Mater. Sci. Eng..

[17] Mohammed W.M., Ng E.G., Elbestawi M.A. (2011). On stress propagation and fracture in compacted graphite iron. Int. J. Adv. Manuf. Technol..

[18] Palkanoglou E.N., Baxevanakis K.P., Silberschmidt V.V. (2021). Thermal debonding in compacted graphite iron: Effect of interaction of graphite inclusions. Procedia Struct. Integr..

[19] Gad S.I., Attia M.A., Hassan M.A., El-Shafei A.G. (2021). Predictive computational model for damage behavior of metal-matrix composites emphasizing the effect of particle size and volume fraction. Materials.

[20] Naghdinasab M., Farrokhabadi A., Madadi H. (2018). A numerical method to evaluate the material properties degradation in composite RVEs due to fiber-matrix debonding and induced matrix cracking. Finite Elem. Anal. Des..

[21] Niu J., Huang C., Shi Z., Liu H., Tang Z., Su R., Chen Z., Li B., Wang Z., Xu L. (2024). A chip formation mechanism taking into account microstructure evolution during cutting process: Taking compacted graphite iron machining as an example. Int. J. Mach. Tools Manuf..

[22] Yang W.J., Pang J., Wang L., Wang S.G., Zhang Z.F. (2021). Tensile properties and damage mechanisms of compacted graphite iron based on microstructural simulation. Mater. Sci. Eng. A.

[23] Zhang Y.Y., Shen R.L., Li M.Z., Pang J.C., Zhang Z.F. (2020). Mechanical damage behavior of metal matrix composites with the arbitrary morphology of particles. J. Mater. Res. Technol..

[24] He Y., Zhang J., Qi Y., Liu H., Memon A.R., Zhao W. (2017). Numerical study of microstructural effects on chip formation in high speed cutting of ductile iron with discrete element method. J. Mater. Process. Technol..

[25] Palkanoglou E.N., Baxevanakis K.P., Silberschmidt V.V. (2022). Thermal debonding of inclusions in compacted graphite iron: Effect of matrix phases. Eng. Fail. Anal..

[26] Haeri M., Haeri M. (2015). ImageJ Plugin for Analysis of Porous Scaffolds used in Tissue Engineering. J. Open Res. Softw..

[27] Johnson G.R., Cook W.H. A constitutive model and data for metals subjected to large strains, high strain rates and high temperatures. Proceedings of the 7th International Symposium on Ballistics.

[28] Mohammed W.M., Ng E., Elbestawi M.A. (2012). Modeling the effect of compacted graphite iron microstructure on cutting forces and tool wear. CIRP J. Manuf. Sci. Technol..

[29] Razanica S., Josefson L.B., Larsson R., Sjögren T. (2021). Validation of the ductile fracture modeling of CGI at quasi-static loading conditions. Int. J. Damage Mech..

[30] Greenstreet W.L., Yahr G.T., Valachovic R.S. (1969). The behavior of graphite under biaxial tension. Carbon.

[31] Joshi A., Baxevanakis K.P., Silberschmidt V.V. (2023). High-temperature creep of cast irons. Adv. Struct. Mater..

